# Metabolic responses to acute physical exercise in young rats recovered from fetal protein malnutrition with a fructose-rich diet

**DOI:** 10.1186/1476-511X-10-164

**Published:** 2011-09-21

**Authors:** Lucieli T Cambri, Gustavo G de Araujo, Ana C Ghezzi, José D Botezelli, Maria AR Mello

**Affiliations:** 1 Department of Physical Education, São Paulo State University (UNESP), Rio Claro-SP, Brazil

**Keywords:** low protein, nutritional recovery, metabolic syndrome, metabolism, physical exercise, maximal lactate steady-state

## Abstract

**Background:**

Malnutrition *in utero *can "program" the fetal tissues, making them more vulnerable to metabolic disturbances. Also there is association between excessive consumption of fructose and the development of metabolic syndrome. However, there is little information regarding the acute effect of physical exercise on subjects recovered from malnutrition and/or fed with a fructose-rich diet. The objective of this study was to evaluate the metabolic aspects and the response to acute physical exercise in rats recovered from fetal protein malnutrition with a fructose-rich diet.

**Methods:**

Pregnant Wistar rats were fed with a balanced (B) diet or a low-protein (L) diet. After birth and until 60 days of age, the offspring were distributed into four groups according to the diet received: B: B diet during the whole experiment; balanced/fructose (BF): B diet until birth and fructose-rich (F) diet afterwards; low protein/balanced (LB): L diet until birth and B diet afterwards; low protein/fructose (LF): L diet until birth and F diet afterwards.

**Results:**

The excess fructose intake reduced the body weight gain, especially in the BF group. Furthermore, the serum total cholesterol and the LDL cholesterol were elevated in this group. In the LF group, the serum total cholesterol and the muscle glycogen increased. Acute physical exercise increased the serum concentrations of glucose, triglycerides, HDL cholesterol and liver lipids and reduced the concentrations of muscle glycogen in all groups.

**Conclusion:**

An excess fructose intake induced some signs of metabolic syndrome. However, protein malnutrition appeared to protect against the short term effects of fructose. In other hand, most responses to acute physical exercise were not influenced by early malnutrition and/or by the fructose overload.

## Background

The worldwide incidence of severe malnutrition has decreased in the last decades. However, malnutrition considered as "mild" or "moderate", associated with anemia of pregnancy, low birth weights and stunted growth in children, is still highly prevalent in third world countries. In 2000, the incidence of children born globally with low birth weight was about 15.5%, with the highest rate (27.1%) observed in South Asia. More than 20 million underweight children are born every year in developing countries [1].

Malnutrition *in utero *can "program" the fetal tissues, making them more vulnerable to disturbances associated with eating, such as type 2 diabetes, metabolic syndrome and other chronic diseases in adulthood [2,3]. Likewise, the excessive consumption of fructose in the diet of contemporary society has interested researchers in the field of public health. There is clinical and epidemiological evidence indicating an association between the excessive consumption of fructose, a sweetener widely used in soft drinks and other foods, and the development of metabolic syndrome [4,5]. Thus, it is relevant to determine whether organisms subjected to early malnutrition are more susceptible to the deleterious metabolic effects of fructose overload in the diet. We hypothesized that fetal protein malnutrition would increase the impact of a fructose-rich diet along metabolic syndrome parameters.

Most studies using animal models examine the influence of early protein malnutrition only on baseline variables, usually after birth or weaning. Likewise, nutritional recovery is often obtained with a balanced diet in the absence of physical exercise [6,7]. In contrast, signs of metabolic syndrome in adult rats, such as hypertension, hyperinsulinemia, hypertriglyceridemia and insulin resistance [8-12] are often triggered by the administration of fructose-rich diets. Therefore, the interactions among nutritional recovery, a fructose-rich diet and the effects of physical exercise are important to elucidate.

Some studies have shown beneficial effects of regular physical exercise on the growth of malnourished children and rats [13,14], indicating that physical exercise can positively impact nutritional recovery. Similarly, although there is still no consensus on the long-term effects of physical exercise on the metabolic syndrome, its benefits are evident in isolated diseases, such as obesity [15], diabetes mellitus [16], dyslipidemia [17] and hypertension [18]. However, there is little information regarding the acute effects of physical exercise on subjects recovering from protein malnutrition and/or being fed a fructose-rich diet. With this, it is important to evaluate some metabolic parameters after acute physical exercise realized in the maximal lactate steady-state, because this intensity can be used to an appropriate exercise prescription in the long-term training, to improve the metabolic parameters associated the metabolic syndrome, so as, to determinate the possible effects of physical training during the nutritional rehabilitation.

Therefore, the objective of this study was to evaluate the metabolic characteristics of young rats recovered from fetal protein malnutrition with a fructose-rich diet, as well as to examine the acute response to physical exercise performed at the maximal lactate steady-state intensity in these animals.

## Materials and methods

### Animals and diets

Twenty four pregnant adult (90 days) Wistar rats were kept in individual cages at a room temperature of 25°C with a photoperiod of 12 hours of light and 12 hours of darkness, with lights on from 06:00 to 18:00 h and with free access to water and food during the entire experiment. All procedures involving the animals were approved by the Committee of Ethics in Animal Research of the State University of Campinas (UNICAMP) under protocol n°1487-1.

The rats were fed with isocaloric (3.766 kcal/g) balanced (17% protein) [19], low-protein (6% protein) [7] and fructose-rich (60% fructose) [11] diets, with the compositions described in Table 1. Body weight of the newborn was registered once a week, from weaning (21 days) until 60 days of age.

**Table 1:** Composition of the diets

Components (g/kg)	**Balanced (17%)**^**1**^	**Low-protein (6%)**^**2**^	Fructose **(60%)**^**3**^
**Casein**^**4**^	202	71.5	202
**Starch**	397	480	-
**Dextrin**	130.5	159	-
**Sucrose**	100	121	27.6
**Fructose**	-	-	600
**L-cystine**	3	1	3
**Soybean oil**	70	70	70
**Mineral mix (AIN-93GMX)**^**1**^	35	35	35
**Vitamin mix (AIN-93GVX)**^**1**^	10	10	10
**Fiber**	50	50	50
**Choline hydrochloride**	2.5	2.5	2.5

^1^According to the American Institute of Nutrition (AIN-93G) - [19]. Diet containing 17% protein.

^2 ^According to Latorraca et al. [7]. Diet containing 6% protein.

^3 ^According to Moura et al. [11]. Diet containing 17% protein and 60% fructose.

^4^Values corrected according to the amount of protein in the casein.

### Experimental groups

According to the diet received during pregnancy (21 days), the mother rats were separated into two groups: a balanced (B) - diet containing 17% protein, and a low protein (L) - diet containing 6% protein.

Immediately after birth, eight male B neonates and eight male L neonates were weighed and killed to determine the serum glucose, total protein, albumin [20], liver glycogen [21] and lipids [20] to confirm the installation of malnutrition in the L group. The remaining male offspring were distributed into four groups (20 rats per group) according to the diet fed until 60 days of age. The litters were adjusted until each female fed eigh offspring. After weaning, the rats were kept in collective cages (five animals per cage). Until weaning (from birth to 21 days), the mothers were fed corresponding diets of the offspring:

• Balanced (B): a diet containing 17% protein during the whole experiment;

• Balanced/Fructose (BF): a diet containing 17% protein until birth and a fructose-rich diet from birth until 60 days of age;

• Low protein/Balanced (LB): a diet containing 6% protein until birth and a diet with 17% protein from birth until 60 days of age;

• Low protein/Fructose (LF): a diet containing 6% protein until birth and a fructose-rich diet from birth until 60 days of age.

### Tissue sample collection

At the end of the experiment, half the rats in each group (10 rats per group) were killed at rest by decapitation 48 hours after the last *in vivo *evaluation. The others were subjected to a single swimming exercise session for 20 minutes, just before euthanasia, while supporting a load equivalent to the maximal lactate steady-state, as described by Gobatto et al. [22]. On alternate days, with a 48-hour minimum interval, different overloads were applied using small bags with lead tied to the chest, which varied from 6.5 to 8.5% of body weight. The maximal lactate steady-state is defined as the highest lactate concentration and workload that can be sustained during physical exercise sessions with fixed loads, without continuous accumulation of blood lactate [23]. The load associated with a maximal lactate steady-state is often used for the prescription of training and the evaluation of aerobic capacity [24].

In the present study, the mean load, corresponding to the maximal lactate steady-state, varied between 7.4 and 7.7% in relation to body weight, and there was no statistically significant difference among the groups. Before undergoing the swimming tests to determine aerobic capacity by the maximal lactate steady-state, the rats were adapted to water for 10 days, once a day, in order to reduce the stress caused by the physical exercise performed in the water. The both water level, the water exposure time and the overload sustained by the rats were subsequently increased. The water temperature was always kept at 31 ± 1°C. Blood samples were collected immediately after killing for serum separation to determine levels of glucose, triglycerides, total cholesterol, LDL cholesterol, HDL cholesterol, total protein and albumin determinations by colorimetric methods using commercial kits (LABORLAB^®^, Guarulhos, São Paulo/Brazil).

The adipose tissue from the mesenteric, retroperitoneal and posterior subcutaneous regions was removed to determine weight and total lipid concentration. Excision of the different fat deposits was carried out according to the description of Cinti [25]. The concentrations of lipids in these deposits were determined by the procedure described by Nogueira et al. [20]. Liver and soleus muscle samples were removed for glycogen concentration determination [21], and liver samples were removed for total lipid determination [20].

### Statistical Analysis

A Student's t-test was used for comparisons between the newborns. For comparisons among groups (intergroup), both at rest and after acute exercise, a two-way ANOVA, followed by a post-hoc Newman-Keuls test when necessary, was used at the end of the experiment. For intra-group comparisons on the effects of acute physical exercise, a Student's t-test was used. A 5% (p < 0.05) level of significance was adopted.

## Results

At birth, the low protein diet caused a statistically significant reduction (p < 0.05) in body weight (B: 6.66 ± 0.79; L: 5.32 ± 0.85 g), serum glucose levels (B: 80 ± 10; L: 67 ± 18 mg/dL), serum albumin (B: 3.37 ± 0.20; L: 1.29 ± 0.19 mg/dL) and serum protein (B: 7.43 ± 1.83; L: 3.95 ± 1.83 mg/dL) and a statistically significant increase (p < 0.05) in the concentrations of liver lipids (B: 7.12 ± 1.69; L: 17.74 ± 5.46 mg/100 g) and liver glycogen (B: 2.02 ± 0.59; L: 2.46 ± 0.22 mg/100 g).

Table 2 presents the body weight gain from weaning to the end of the experiment and the weight of adipose tissue from different anatomical regions by the end of the experiment. It is evident that the weight gain was influenced by malnutrition and excessive fructose consumption because all other groups showed significant differences among themselves (p < 0.05). The group that received the fructose-rich diet after birth (BF) had the lowest body weight gain compared to the others, followed by the group that also received the fructose-rich diet after birth but was malnourished during the fetal period (LF). Similarly, the weight of the subcutaneous adipose tissue was influenced by the malnutrition and by the excess fructose in the diet, because all the groups had lower weights of adipose tissue in this region than did the controls (B).

**Table 2:** Body weight gain from weaning (21 days) until the end of the experiment (60 days), weight and total lipids concentrations of the adipose tissue from different anatomical regions at the end of the experiment (60 days)

	B	BF	LB	LF
**Body weight gain (g)**	161.4 ± 25.2^b^	103.4 ± 29.6^d^	186.1 ± 29.8^a^	134.5 ± 22.9^c^
**Weight of the mesenteric adipose tissue (mg/100 mg)**	0.57 ± 0.14	0.45 ± 0.17	0.48 ± 0.10	0.55 ± 0.11
**Weight of the retroperitoneal adipose tissue (mg/100 mg)**	0.35 ± 0.13	0.25 ± 0.06	0.38 ± 0.13	0.37 ± 0.09
**Weight of the subcutaneous adipose tissue (mg/100 mg)**	0.55 ± 0.08^a^	0.40 ± 0.10^b^	0.42 ± 0.07^b^	0.39 ± 0.13^b^
**Lipids of the mesenteric adipose tissue (mg/100 mg)**	70.23 ± 15.60	59.57 ± 13.90	64.32 ± 17.09	75.42 ± 17.73
**Lipids of the retroperitoneal adipose tissue (mg/100 mg)**	21.35 ± 8.72^b^	35.59 ± 9.78^a^	31.36 ± 8.78^ab^	28.67 ± 6.68^ab^
**Lipids of the subcutaneous adipose tissue (mg/100 mg)**	38.74 ± 9.77	37.32 ± 5.24	33.67 ± 7.25	31.26 ± 3.38

Results expressed as the mean ± standard deviation of 10 rats per group.

B: balanced; BF: balanced/fructose; LB: low protein/balanced; LF: low protein/fructose.

Different letters indicate significant difference among groups. Two-Way ANOVA and Newman-Keuls' Post-Hoc (p < 0.05).

Regarding the lipid concentrations in the adipose tissue at the end of the experiment (Table 2), only the BF group showed higher values when compared with the controls (B) for the retroperitoneal region.

**Figure 1:**
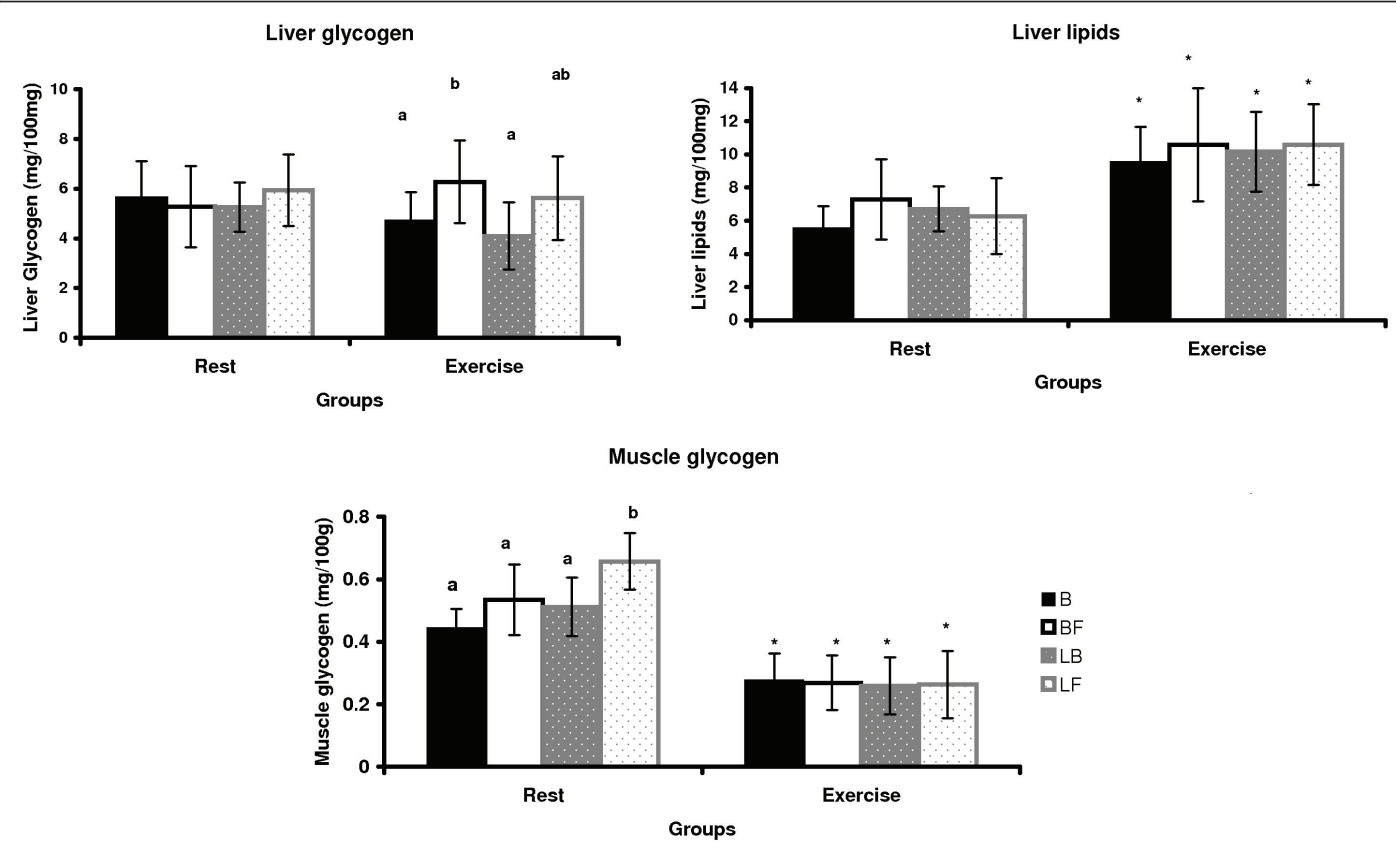
**Tissue variables (mg/100 g) at rest and after acute physical exercise at the end of the experiment (60 days)**. Results expressed as the mean ± standard deviation of 10 rats per group. B: balanced; BF: balanced/fructose; LB: low protein/balanced; LF: low protein/fructose. Different letters indicate significant difference among groups. Two-Way ANOVA and Newman-Keuls' Post-Hoc (p < 0.05). *intra-group difference by Student's t-test (at rest *vs*. acute physical exercise).

Table 3 and Figure 1 show that at 60 days, the serum total protein and albumin, liver glycogen and lipids, which were altered at birth due to fetal malnutrition, were restored, regardless of the diet during the nutritional recovery. However, the concentrations of serum glucose were not fully restored with the fructose-rich diet after fetal malnutrition (LF). The lipid profile was changed in the BF group because the total cholesterol and LDL cholesterol were higher in this group (p < 0.05) in comparison to the B group. However, in the LF group, there was a statistically significant increase (p < 0.05) only in total cholesterol. Triglycerides were higher (p < 0.05) in the BF group compared to the LF group.

**Table 3:** Serum variables at rest (R) and after acute exercise (E) at the end of the experiment (60 days)

		B	BF	LB	LF
**Glucose (mg/dL)**	**R**	107 ± 15^a^	110 ± 9^a^	106 ± 19^a^	83 ± 25^b^
	**E**	126 ± 31*	134 ± 26*	148 ± 35*	152 ± 26*
**Triglycerides (mg/dL)**	**R**	61 ± 13^ab^	79 ± 12^a^	66 ± 28^ab^	53 ± 23^b^
	**E**	95 ± 25*	106 ± 30*	110 ± 22*	104 ± 29*
**Total cholesterol (mg/dL)**	**R**	67 ± 14^b^	124 ± 21^a^	89 ± 26^b^	119 ± 27^a^
	**E**	81 ± 13^b^*	105 ± 14^a^*	82 ± 16^b^	97 ± 26^ab^
**HDL cholesterol (mg/dL)**	**R**	39 ± 6^b^	47 ± 4^a^	36 ± 4^b^	39 ± 8^b^
	**E**	50 ± 5^ab^*	54 ± 6^a^*	45 ± 4^b^*	50 ± 7^ab^*
**LDL cholesterol (mg/dL)**	**R**	59 ± 14^a^	75 ± 14^b^	53 ± 15^a^	50 ± 16^a^
	**E**	58 ± 9	57 ± 11*	49 ± 12	53 ± 10
**Total Proteins (g/dL)**	**R**	5.5 ± 0.1^b^	5.6 ± 0.1^b^	5.5 ± 0.3^b^	5.8 ± 0.2^a^
	**E**	5.6 ± 0.1*	5.7 ± 0.1	5.7 ± 0.1	5.7 ± 0.1
**Albumin (g/dL)**	**R**	3.9 ± 0.3^b^	3.8 ± 0.2^b^	4.2 ± 0.3^a^	4.1 ± 0.6^a^
	**E**	3.9 ± 0.2	3.9 ± 0.2	3.9 ± 0.2*	3.9 ± 0.3

Results expressed as the mean ± standard deviation of 10 rats per group.

B: balanced; BF: balanced/fructose; LB: low protein/balanced; LF: low protein/fructose.

R: at rest; E: after acute physical exercise.

Different letters indicate significant difference among groups. Two-Way ANOVA and Newman-Keuls' Post-Hoc (p < 0.05).

*intra-group difference by Student's t-test (at rest *vs*. acute physical exercise).

The acute physical exercise increased (p < 0.05) the concentrations of serum glucose, triglycerides and HDL cholesterol in all groups (intra-group), but this increase was not different among the groups (intergroup). The total cholesterol changed (p < 0.05) after the physical exercise only in the groups that ingested the B diet during pregnancy (B and BF), and the LDL cholesterol decreased (p < 0.05) only in the BF group.

There were no differences among the groups in liver glycogen concentrations at rest (Figure 1). Additionally, physical exercise did not alter this variable compared to resting values. However, in the BF group, the physical exercise increased the liver glycogen values (p < 0.05) compared to the groups that ingested the balanced diet after birth (B and LB).

The liver total lipids did not differ among the groups, both at rest and after acute physical exercise. However, acute physical exercise significantly increased (p < 0.05) the concentrations of liver lipids in all groups compared with the resting concentrations.

The fructose-rich diet after fetal protein malnutrition (LF) increased (p < 0.05) the concentrations of glycogen in the soleus muscle. However, the acute physical exercise reduced (p < 0.05) the concentrations of muscle glycogen in all groups, which were otherwise not different among themselves after the exercise regimen.

## Discussion

The aim of this study was to evaluate the metabolic aspects of rats recovered from fetal protein malnutrition with a fructose-rich diet and to analyze the acute metabolic responses to a single episode of swimming exercise performed at the maximal lactate steady-state intensity. This is because, oftentimes the studies on the metabolic effects of physical exercise in animal models are criticized due to the lack of information about the intensity of effort performed during the physical exercise.

The low protein diet used in this study proved effective in inducing protein malnutrition during the fetal period, as it reduced body weight, serum glucose, albumin and total protein and increased liver lipids and glycogen in the newborn rats. These findings are similar to those found in several studies that employed a similar procedure for protein malnutrition induction [7,14,26,27].

In accordance with some studies reported in the literature that used a balanced diet for nutritional recovery [6,7,28], in the present study, restoration of the serum variables (total protein and albumin) as well as the tissue variables (liver glycogen and lipids) was observed independent of the diet employed for the nutritional recovery. In other hand, serum glucose did not normalize in the LF group, indicating only a partial efficacy of the fructose-rich diet in the ability to recover from malnutrition.

The rats that consumed the fructose-rich diet had lower body weight gain, especially those who were not malnourished in the fetal period (BF), suggesting that intrauterine protein malnutrition attenuated the body weight impairment caused by fructose. Several studies show that body weight does not differ between rats that consume a balanced diet or a fructose-rich diet [9,11,29-31]. The reduction in body weight gain observed in this study may be due to fructose intolerance because an excess of this component in the diet was imposed at a crucial stage (neonatal) of life. Recently, it was showed that, under normal conditions, intestinal GLUT5 (the fructose transporter at the apical cell membrane) mRNA levels and fructose transport rates are very low in the suckling periods of rat development [32]. In these cases, removing fructose from the diet seems essential to reduce the damage it has caused [33]. The values of weight of adipose tissue in different regions can did not have altered in according body weight because are relative to body weight and not absolute values.

In a previous study with an identical experimental model [12] (except for a longer exposure to the diet - until adulthood), the same impairment in body weight gain was observed in rats that ingested fructose-rich diets. However, aside from the study noted, there is no other report in the literature describing animal growth impairment imposed by early consumption of fructose and no studies using fructose-rich diets administered from the weaning period until adulthood or from birth. Therefore, further studies on this subject are of extreme importance.

Fructose was used in the nutritional recovery to verify whether its effect on metabolic syndrome parameters was increased after fetal malnutrition. Fructose is considered to be more lipogenic than glucose [31]. Therefore, its high intake is often implicated in high levels of plasma triglycerides [9,11,12,29,30,34]. Some mechanisms may account for this relationship, including increased hepatic lipogenesis and a high production of very low density lipoprotein (VLDL) [4]. Fructose metabolism in the liver surpasses the regulatory step of glycolysis catalyzed by the phosphofructokinase. Therefore, fructose continuously enters the glycolytic pathway at the glyceraldehyde-3-phosphate and dihydroxyacetone phosphate level. Both are intermediates in triglycerides synthesis [29,30]. So, this can be a cause of dyslipidemia by excess in the fructose consumption.

In other hand, the effects of fructose on the metabolism of total cholesterol are contradictory, with some studies showing an increase in total serum cholesterol [9,12,29] but others not [11]. In the LF group, serum triglycerides and LDL cholesterol probably did not increase due to the short duration of exposure to the diet. By contrast, in a previous experiment conducted by our research group, keeping the rats on the high fructose diet until 90 days led to elevations in serum triglycerides for both the BF and LF groups [12]. This finding raises the hypothesis that protein malnutrition exerts a protective effect against the development of signs of metabolic syndrome in a short-term exposure to a high fructose diet. One explanation is that, as we studied young animals (60 days of age), probably, they were still in a stage of recovery from malnutrition, ie, first there was a stabilization of the parameters affected by intrauterine malnutrition in relation the control group, and after that, the elevation of these parameters. With this, the ideal would be to conduct a study with analysis of these parameters in different ages, young rat, young adult rats and mature adult rats.

Similar to some of the results of this study, serum concentrations of glucose, free fatty acids [35,36], total cholesterol [35], HDL cholesterol [37] and corticosterone [35,36] are increased, and serum insulin and muscle glycogen are decreased [35,36] after a single session of swimming exercise in rats [35,36] and of treadmill exercise in humans [37]. The hyperglycemic response observed is probably associated with increased hepatic glycogenolysis resulting from the increased sympathetic activity or with the increased gluconeogenesis from the increased glucocorticoid secretions such as corticosterone in animals [35,36] and cortisol in humans [38]. This response may also be related to the increased secretion of the other counter-regulatory hormones such as growth hormone and glucagon after acute physical exercise [38-40], both of which are responsible for the increased availability of glucose to the active muscles.

Differences among the results of some studies may be related to the different experimental protocols and characteristics of the animals and/or subjects evaluated, which include modality, intensity and duration of the acute physical exercise session as well as the age, baseline lipid profile and fitness of the individuals evaluated.

The reduction in the concentrations of muscle glycogen is associated with increased muscle glycogenolysis by the acute increase of catabolic hormones [34,36,39,40]. It also depends on the intensity of the physical exercise because, as other studies show, the reduction of muscle glycogen is sharper at 76 than at 48% VO_2 max _[41]. In the present study, the data suggest an increased use of muscle glycogen and lower lipids utilization, corroborating the idea that high intensity physical activity is related to increased muscle glycogen depletion because the acute physical exercise was performed at the intensity of the maximal lactate steady-state.

Exercise time (20 minutes) in the present study also explains the increased use of carbohydrates because, according to Schrauwen-Hinderling et al. [42], this metabolism is predominant at 30 minutes of physical exercise at 55% VO_2 max_. In the latter study, there was an incremental increase in intramuscular lipids in inactive muscles compared to active muscles during physical exercise due to the greater availability of plasma free fatty acids, with a concomitant increase in the uptake of free fatty acids by the muscle tissue [42]. This result may explain the increase in liver lipids after physical exercise observed in this study. Because the release of free fatty acids by lipolysis of the adipose tissue exceeds the oxidation capacity of active muscles during prolonged physical exercise [43], the remaining free fatty acids can be re-esterified in the adipose tissue, in active or inactive skeletal muscle and in the liver [42].

In summary, excess fructose intake reduced the body weight gain in rats, particularly those that were not malnourished during the fetal period (BF). Moreover, the high serum concentrations of total cholesterol and LDL cholesterol observed in this group (BF) are indicators of the development of some signs of metabolic syndrome, unlike the group that consumed fructose after protein malnutrition (LF), in which only serum total cholesterol increased. Therefore, protein malnutrition appeared to protect against the short term effects of fructose. These observations contradict the hypothesis that early malnourished organisms are more susceptible to the deleterious metabolic effects of excess fructose in the diet.

Regarding the effects of acute physical exercise, most of the metabolic responses were similar for all groups, regardless of the early protein malnutrition and the high fructose diet. This suggest that the intensity of physical exercise utilized is secure, from the metabolic point of view and can be used in long-term training to improve the metabolic parameters initially altered by the experimental model used. These data indicate the importance of further research, aiming to clarify questions that are still obscure about the metabolic effects of the early excessive consumption of fructose and of long term physical training.

## List of Abbreviations

B: balanced diet; BF: balanced/fructose diet; F: fructose diet; L: low protein diet; LB; low protein/balanced diet; LF: low protein/fructose diet.

## Competing interests

The authors declare that they have no competing interests.

## Authors' contributions

LTC was responsible for the experimental design, data collection and preparation of the manuscript. GGA, ACG and JDB were responsible for the data collection and the preparation of the manuscript. MARM was responsible for experimental design, coordination of research and preparation of the manuscript. All authors read and approved the final manuscript text.
